# Does health information technology improve acknowledgement of radiology results for discharged Emergency Department patients? A before and after study

**DOI:** 10.1186/s12911-020-01135-9

**Published:** 2020-06-03

**Authors:** Julie Li, Richard Paoloni, Ling Li, Joanne Callen, Johanna I. Westbrook, William B. Runciman, Andrew Georgiou

**Affiliations:** 1 Centre for Health Systems and Safety Research, Australian Institute of Health Innovation, Macquarie University, Level 6, 75 Talavera Rd, Sydney, NSW 2109 Australia; 2 Emergency Department, Concord Repatriation General Hospital, Sydney, Australia; 3 Sydney Medical School, University of Sydney, Sydney, Australia; 4 Australian Centre for Precision Health, Cancer Research Institute, University of South Australia, Adelaide, Australia; 5 Australian Patient Safety Foundation, Adelaide, Australia

**Keywords:** Medical informatics, Radiology, Medical errors, Duty to recontact, Evaluation studies

## Abstract

**Background:**

The inadequate follow-up of test results is a key patient safety concern, carrying severe consequences for care outcomes. Patients discharged from the emergency department are at particular risk of having test results pending at discharge due to their short lengths of stay, with many hospitals acknowledging that they do not have reliable systems for managing such results. Health information technology hold the potential to reducing errors in the test result management process. This study aimed to measure changes in the proportion of acknowledged radiology reports pre and post introduction of an electronic result acknowledgement system and to determine the proportion of reports with abnormal results, including clinically significant abnormal results requiring follow-up action.

**Methods:**

A before and after study was conducted in the emergency department of a 450-bed metropolitan teaching hospital in Australia. All radiology reports for discharged patients for a one-month period before and after implementation of the electronic result acknowledgement system were reviewed to determine; i) those that reported abnormal results; ii) evidence of test result acknowledgement. All unacknowledged radiology results with an abnormal finding were assessed by an independent panel of two senior emergency physicians for clinical significance.

**Results:**

Of 1654 radiology reports in the pre-implementation period 70.6% (*n* = 1167) had documented evidence of acknowledgement by a clinician. For reports with abnormal results, 71.6% (*n* = 396) were acknowledged. Of 157 unacknowledged abnormal radiology reports reviewed by an independent emergency physician panel, 34.4% (*n* = 54) were identified as clinically significant and 50% of these (*n* = 27) were deemed to carry a moderate likelihood of patient morbidity if not followed up. Electronic acknowledgement occurred for all radiology reports in the post period (*n* = 1423), representing a 30.4% (95% CI: 28.1–32.6%) increase in acknowledgement rate, and an increase of 28.4% (95% CI: 24.6–32.2%) for abnormal radiology results.

**Conclusions:**

The findings of this study demonstrate the potential of health information technology to improve the safety and effectiveness of the diagnostic process by increasing the rate of follow up of results pending at hospital discharge.

## Background

Health services are prone to preventable medical errors, which can have serious and even fatal consequences [1–3]. Developments in health information technology (IT) present enormous potential to improve health care delivery processes and reduce errors [4–6]. The test management process is one example where the introduction of technology may be helpful [7–10] to reducing errors. Previous research has shown that failure to follow up test results ranges from 20 to 62% of tests for hospital inpatients, and up to 75% for Emergency Department (ED) patients, with subsequent outcomes of missed diagnoses and inappropriate treatment [11].

The transition period after discharge from hospital is especially vulnerable to errors due to communication breakdowns between hospital and general practice providers [12]. Patients discharged from an ED are at particular risk of having test results pending at discharge due to their short lengths of stay [13]. Many hospitals do not have reliable systems for managing test results pending at discharge, and there is some evidence that physician management and awareness of such results are poor [14].

Numerous health IT applications across the US and Australia have been developed to support test result management processes, including systems that can track pending test results at hospital discharge [15], deliver result notifications to clinicians [16] and utilise tracking systems to document test result acknowledgement and subsequent clinical actions [16]. Nevertheless, research continues to point to challenges with the use of electronic test result management systems [17]. Problems include the potential for information overload given the volume of tests requiring acknowledgement [18], negative effects on the efficiency of work [19, 20] and challenges in establishing appropriate escalation procedures to ensure effective test result follow-up [21].

The implementation of a commercially available electronic result acknowledgement (eRA) system in hospitals across the state of New South Wales, Australia, provided an opportunity to investigate the impact of an online process for test acknowledgement in an Emergency department. Missed and delayed diagnoses due to delays in radiology reporting constitute a substantial proportion of medical errors and consequent adverse events in ambulatory patients [5]. A key indicator of the effectiveness of eRA systems is whether they can reduce the problem of abnormal test results not reviewed.

We aimed to measure changes in the proportion of acknowledged radiology reports pre and post the introduction of an eRA system and to determine the proportion of reports with abnormal results, including clinically significant abnormal results requiring follow-up action.

## Methods

### Study design and setting

A before and after study was conducted in the ED of a 450-bed metropolitan teaching hospital in Australia. Characteristics of the study site are presented in Table 1 below.

**Table 1:** Characteristics of study site

Characteristics^a^	Study Site
Hospital Beds	450
Emergency Department (ED) beds	40
Annual ED attendances	58,483
Annual ED discharges	40,700

^a^ All statistics reported over 2012–2013

### Intervention

The study site used a commercial electronic medical record system (eMR), Cerner PowerChart, for ordering and viewing of diagnostic tests for all patients. The online test verification component of the Cerner PowerChart test management system, Message Centre, was implemented at the site in August 2013. Message Centre is an electronic inpatient and outpatient workflow management module comprised of an Inbox containing documents and notifications requiring review, attention, or signature, including all radiology result reports for patients attending the ED. Results arrive electronically and are presented in a main list on a dedicated page in the Message Centre. Once a radiology report is opened, electronic acknowledgement occurs by a clinician clicking on a relevant button at the base of the report, whereby the report disappears from the main list of results. A separate forwarding action can be used to document reminders or handover information to other physicians in instances where follow-up action was not finalised during one particular shift. In such instances, the result would not be acknowledged, and remain in the list. At the time of the study, a concurrent paper medical record was in use in parallel to the eMR.

Prior to system implementation, manual results acknowledgement was completed by the senior emergency physicians on an ad-hoc basis when abnormal results were faxed or communicated verbally by telephone to clinical staff in the ED at the discretion of the ancillary departments. Following eRA system implementation, results of all diagnostic tests ordered from the ED were required to be acknowledged electronically. This was performed by senior emergency physicians on a rotational basis during dedicated daily results acknowledgement sessions as an administrative task.

### Study population

Radiology reports were extracted for all patients who presented to the ED and were subsequently discharged or died during the month of April 2013 prior to eRA system implementation, and in April 2014, eight months following system implementation (August 2013). Discharged patients were defined as patients not requiring hospital admission, or transfer to another hospital.

### Data collection

All radiology examinations for discharged patients who had presented during the pre-period were extracted from the eMR database by IT staff. Read-only access to electronic patient records was granted to researchers who reviewed results to determine; i) those that reported abnormal results and those that showed no abnormalities; ii) evidence of test result acknowledgement. This was recorded on a data collection form co-designed with senior clinicians involved in regular radiology result acknowledgement (see Additional file 1).

A pilot phase involving two researchers (JL, JC) who reviewed a 1% random sample of the pre-implementation data set took place prior to data collection to develop a coding classification to inform the interpretation and categorisation of results for the purposes of the study. Radiology reports were categorised as abnormal by one researcher (JL) if active/current abnormalities of any kind are reported, regardless of clinical significance.

In the pre-implementation period, acknowledgement of radiology test results was determined by one researcher (JL) from; i) documented acknowledgement in electronic notes (e.g. discharge summary), ii) a doctor’s signature or initials on the paper report, or iii) notes referring to the test results in the patient’s hard copy progress notes. In the post-implementation study period, radiology reports were extracted from the eMR and reviewed by the same researcher for categorisation as normal or abnormal. Evidence of electronic result acknowledgement was identified in the post-implementation period by electronic labelling of the result report as “Acknowledged” in the test result verification system.

Reliability of data abstraction from records, and application of the classification was measured using inter-rater reliability testing between two researchers using 5% of pre-implementation data. Kappa scores were calculated to measure: 1) agreement between researchers on results: 0.59 (95% CI: 0.44–0.74); and 2) agreement on acknowledgement: 0.70 (95% CI: 0.56–0.86). These results indicated substantial agreement between the two researchers.

### Assessment of abnormal test results for clinical significance

Abnormal test results were defined as clinically significant if they were judged to have the potential to lead to patient morbidity. All unacknowledged radiology results with an abnormal finding were reviewed by a panel of two senior emergency physicians from a hospital external to the study site. These physicians were asked to review the clinical significance of each abnormal radiology result against clinical benchmarks applied in regular practice and to indicate if the results would have influenced patient management and required action. These reviewing physicians were not provided with any information about the aims of the study and thus were blinded to the study site, intervention (eRA) and the purpose of the study. Physicians were asked to record responses to the following questions:

1. Whether they would review notes for further clinical information (yes/no)
2. What actions need to be taken (free text)
3. The *likelihood* of patient morbidity if result was missed (low, moderate, high)
4. The clinical *severity* of morbidity if result was missed (low, moderate, high)

In cases of disagreement, a third external senior emergency physician reviewed the record and provided a final adjudication. Unacknowledged, clinically significant, abnormal results of a moderate to high *likelihood* of patient morbidity *and* moderate to high *severity* of morbidity if missed were reported back to the study site for follow-up.

### Measurements

Data collected before and after eRA implementation as described in the Data Collection section was linked with patient demographics and hospital admission and discharge date/time using patient medication record number and test date/time ordered. The numbers of unacknowledged radiology report results and unacknowledged abnormal results as a proportion of all radiology reports for patients presenting during the month of April immediately before and after eRA system implementation were calculated. These percentages were stratified by result type (normal vs abnormal). 95% intervals and binomial tests were performed. Level of significance was set at 0.05. The statistical package SAS version 9.4 was used for data manipulation and analysis.

## Results

### Rate of radiology result acknowledgement before eRA system implementation

**Fig. 1:**
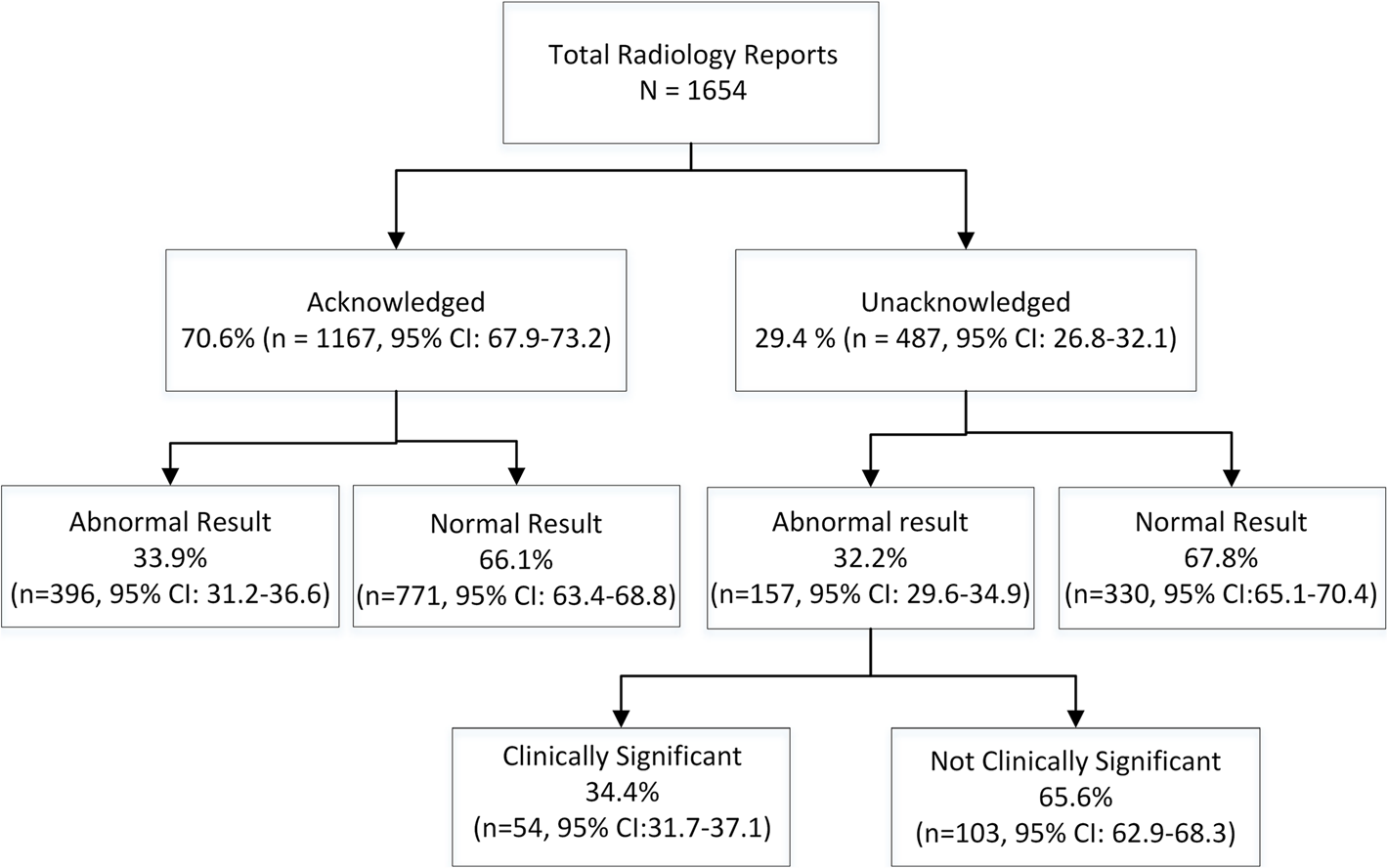
Result acknowledgement pre-eRA implementation

A total of 1654 radiology reports were included in the pre-implementation period. There was evidence of acknowledgement for 70.6% (*n* = 1167, 95% CI: 67.9–73.2) of these radiology results (Fig. 1). Evidence of acknowledgement was identified for the majority (71.6%; *n* = 396, 95% CI: 69.0–74.2) of abnormal results (*n* = 553). Table 2 shows the location of evidence of result acknowledgement in patients’ records. Physicians documented result acknowledgement most frequently in electronic discharge summaries (95.6%).

**Table 2:** Location of radiology result acknowledgement documentation by ED physicians

Documentation of Radiology Result Acknowledgement	N (%)
Hard copy radiology report	9 (0.8)
Progress notes (paper medical record)	7 (0.6)
Electronic discharge summary	1116 (95.6)
Electronic ED Case History notes (in eMR)	26 (2.2)
Soft copy report (in eMR)	5 (0.4)
Other	4 (0.3)
Total	1167

### Clinically significant abnormal radiology results before eRA system implementation

All unacknowledged abnormal radiology reports (*n* = 157) were forwarded to the independent emergency physician panel for review. A total of 34.4% (n = 54) were identified as clinically significant. Of these, half (*n* = 27) were deemed to carry a moderate likelihood of patient morbidity if results were missed. Of these results, 21 were judged to potentially cause patient morbidity of moderate to high severity. No unacknowledged results were considered highly likely to lead to patient morbidity. Table 3 details the outcome of the review including direct extracts from clinically significant abnormal reports.

**Table 3:** Classification of the likelihood and severity of morbidity of unacknowledged significant abnormal radiology reports

Likelihood of Morbidity (N)	Severity of morbidity (N)	Direct extract from radiology report
**Low (27)**	Low (20)	Left mid zone air space infiltrate noted. The remainder of the lungs are clear. No significant pleural effusion bilaterally. Suggest progress imaging.
	Mod (5)	There is impression of superior mediastinal widening. This should be assessed further with departmental imaging, alternatively, comparison with previous imaging is recommended. No other significant finding of interest.
	High (2)	There is an irregular rounded opacity in the left upper lobe. This may represent an area of focal consolidation but underlying mass lesion must be considered and follow-up imaging to resolution is recommended. Attention ED director
**Mod (27)**	Low (6)	Ankle mortise is preserved. There is an acute posterior malleolar fracture. Soft tissue swelling noted around the ankle joint.
	Mod (16)	There is a joint effusion and a nondisplaced radial head fracture is suspected.
	High (5)	Cardiac mediastinal contours are within normal limits. There is a nodular opacity in the left lower lobe, measuring 2.2 × 1.4 cm in size. This may represent a primary or secondary pulmonary neoplastic lesion. The remainder of the lungs are clear. No significant pleural effusion bilaterally. Cardiac mediastinal contours are within normal limits.
**High (0)**	N/A	N/A

### Changes in the rate of radiology result acknowledgement following eRA system implementation

**Fig. 2:**
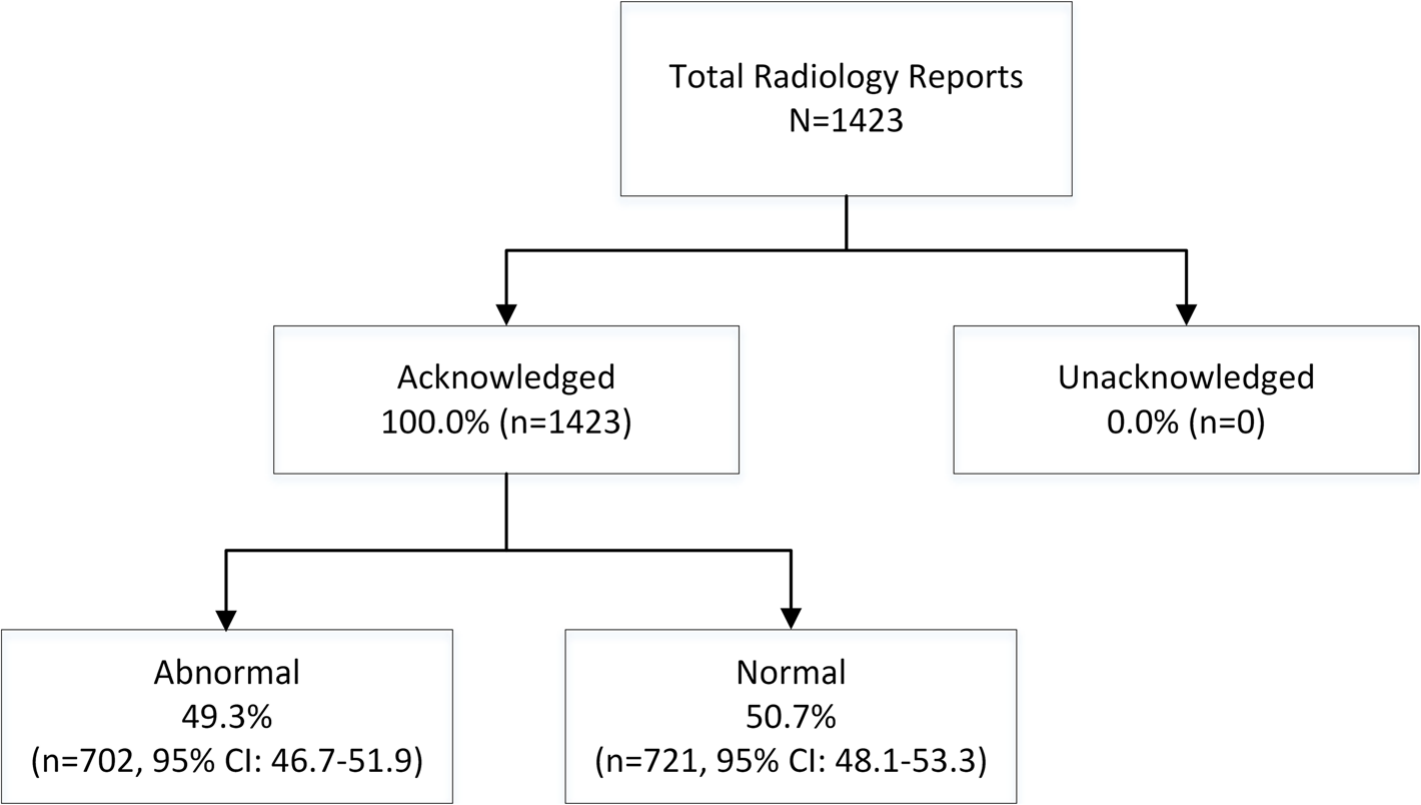
Result acknowledgement post-eRA implementation

Radiology report acknowledgement rates improved significantly (*p* < 0.001) following the implementation of the eRA system (Table 4). Electronic acknowledgement occurred for all radiology results in the post-implementation period (100.0%; *n* = 1423) (Fig. 2). This was a 30.4% (95% CI: 28.1–32.6%) increase in the acknowledgement rate, and an increase of 28.4% (95% CI: 24.6–32.2%) for abnormal radiology results in comparison to the pre-implementation period.

**Table 4:** Abnormal radiology result acknowledgement rates before and after eRA implementation

Study Period	Total no. of abnormal test results	No. of results acknowledged (%; 95% CI)
**Pre eRA**	553	396 (71.6; 67.8–75.4)
**Post eRA**	702	702 (100.0; 100.0–100.0)

## Discussion

Our results showed that nearly 30% of all radiology test results were unacknowledged in the pre-implementation period, consistent with previous studies in the ambulatory setting [22]. This study, however importantly identified that 32% of these tests which were unacknowledged results, and that 50.0% of these were assessed as likely to lead to patient morbidity (of moderate or high severity). Following introduction of a mandatory eRA system, all radiology reports were acknowledged. This represented a 30.4% improvement in the acknowledgement rate overall, and an increase of 28.4% for abnormal radiology results. These results are among the few internationally to quantify changes in acknowledgement rates following eRA system introduction.

eRA provides clinicians and hospital management with a means by which to track and monitor unacknowledged test results. The 100% test result acknowledgement rate found in our study is mirrored by an earlier Australian study [21] which found that the introduction of a mandatory eRA system led to the acknowledgement of all laboratory and medical imaging results. Our results also demonstrated the capacity of the eRA system to standardise the acknowledgement process, centralising documentation of acknowledgement from a number of potential locations to one location that is easily auditable.

However, while clearly demonstrating an improvement in the rate at which physicians indicated that they have viewed radiology results, a limitation of the study is that it is unclear whether electronic acknowledgement resulted in subsequent improvements in patient care. Previous studies have indicated that issues exist which can undermine the intended use and outcomes of eRA. In a US study [23] on the rate of physician use of a voluntary eRA system, authors reported that only 78% of nonurgent clinically significant results were acknowledged electronically. Among the reasons provided for the inconsistent rate of use were the suboptimal integration of the eRA system with workflow and other clinical information systems. Further, there is evidence that indicates that electronic acknowledgement does not necessarily lead to the initiation of appropriate follow-up. Studies which evaluated the impact of a critical result alerting system found that timely follow-up action was equally lacking for both acknowledged and unacknowledged results [24, 25]. eMR-based, individual level tracking of follow-up actions has been recommended to ensure “genuine” acknowledgement of results [25]. This includes, for example, physicians specifically selecting from a drop-down menu of follow-up actions for each result in the eRA system (e.g., contact patient, no further action etc.), to document actions taken following review of the result. These actions could then be tracked and reviewed as part of a regular reporting process.

The acknowledgement of test results represents one facet of a complex process, and the introduction of information technology is not a complete solution to the problem. Alongside systems implementation is the need for clearer lines of test follow-up responsibility and escalation procedures, and fostering of staff engagement through invitation for feedback about experiences of system use [21]. Deliberations regarding the ability to integrate interventions within clinical workflow and across hospital electronic systems should also be made to minimise risks of work practice change and achieve intended use of systems [23].

The findings from this study illustrate the potential of health IT to improve the safety and effectiveness of the test results follow up process. This study did not adopt a controlled study design, and therefore results are unable to be adjusted for potential confounding variables including changes in the makeup of the physician workforce, patient casemix and types of test results reviewed.

## Conclusions

The important patient safety problem of delayed or incomplete test result follow-up remains an area of ongoing concern [26]. The diagnostic process involves a series of related information sharing activities including the gathering, interpretation and transfer of information to clinicians, patients and management [27]. Results acknowledgement functions can contribute to greater transparency and accountability of test result management and help to ensure that all test results are reviewed as a prerequisite to organising appropriate follow-up care and to potentially improve patient outcomes.

## Supplementary information

**Additional file 1.** Data collection form.

## Data Availability

Conditional and restricted access to relevant Local Health District data used and analysed during this study was granted under ethical approval. Local Health District data are not publicly available.

## Abbreviations

IT: Information technology
ED: Emergency department
eRA: Electronic results acknowledgement
eMR: Electronic medical record
