# Supplementary figures and images for: Does health information technology improve acknowledgement of radiology results for discharged Emergency Department patients? A before and after study
Source: BMC Med Inform Decis Mak. 2020 Jun 3;20:100. doi: 10.1186/s12911-020-01135-9 (PMC7268495; doi:10.1186/s12911-020-01135-9)

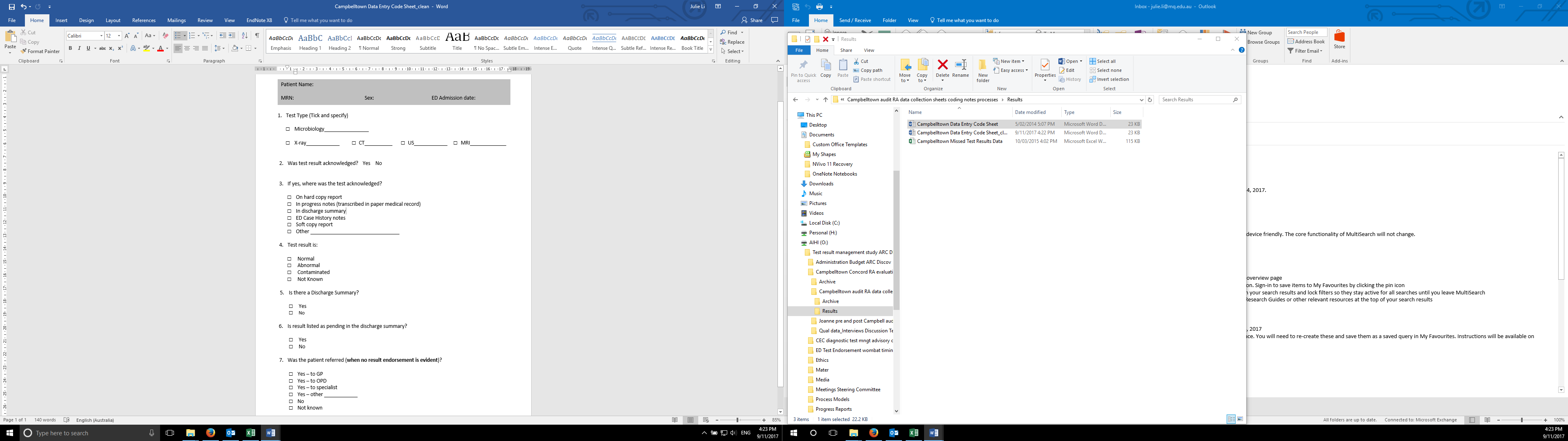
**Additional file 1 - Data Collection Sheet**

Supplement: Supplementary file 1 — Additional file 1. Data collection form. [file 12911_2020_1135_MOESM1_ESM.docx]
